# Commentary: Bettering BCG: a tough task for a TB vaccine?

**DOI:** 10.3389/fimmu.2019.02195

**Published:** 2019-09-12

**Authors:** Brahm S. Srivastava, Vipul K. Singh, Vivek K. Kashyap, Ranjana Srivastava, Arshad Khan, Chinnaswamy Jagannath

**Affiliations:** ^1^Nextec Lifesciences, Lucknow, India; ^2^Department of Pathology and Genomic Medicine, Houston Methodist Research Institute, Houston, TX, United States; ^3^Department of Immunology and Microbiology, University of Texas Rio Grande Valley, McAllen, TX, United States

**Keywords:** LipY lipase from *Mycobacterium tuberculosis*, BCG—bacille calmette-guérin vaccine, *M. tuberculosis*, vaccine, booster antigen

The BCG (Bacille Calmette-Guerin) vaccine is the only licensed vaccine for tuberculosis (TB), with several million children vaccinated to date. BCG protects children against disseminated TB, but shows variable efficacy in adults against pulmonary TB. Multiple efforts have been undertaken to improve BCG but have had unsatisfactory results, although these variations have largely been tested only in animal models of TB. These efforts have included: (i) recombinant BCG (rBCG) expressing cytokines, (ii) putative protective antigens derived from *Mycobacterium tuberculosis*, and (iii) subunit antigens purported to add immunogenic “value” to BCG (1).

In an attempt to improve BCG, a modified vaccinia virus Ankara expressing antigen 85A (MVA85A) was administered to previously BCG-vaccinated infants and young children in a clinical trial conducted in South Africa. Investigators were hopeful based on promising results observed in animal models of TB. However, the booster vaccine failed to protect vaccine recipients from infection with *M. tuberculosis* (2). Bishai et al. expressed disappointment with the outcome of the trial (3). Nevertheless, they expressed hope and proposed some issues for future consideration. The results of this trial emphasize the need for identification of biomarkers that correlate with protection and active TB in young and adult human subjects, which should be the gold standard by which the efficacy of new vaccines are evaluated. Selection of young BCG-vaccinated children to test the efficacy of MVA85A has also been questioned. Since, TB is a lung disease that occurs in the adult population, conducting a trial for booster vaccines may be more relevant in BCG-vaccinated adolescents (15+ years old). Bishai et al. propose to conduct any future trials of booster vaccines in adolescent and/or adult population, infected with active or latent *M. tuberculosis* (3), especially in Indian and African continents where TB is endemic. The logic of selection of modified vaccinia virus as a carrier of antigen 85A is also intriguing, since the world's population has received smallpox vaccinations. Whether the engineered vaccinia vaccine MVA85A will be able to survive and replicate in a vaccinated population long enough to induce sufficient immunity is questionable.

Despite these setbacks, there are new candidates in the pipeline currently being evaluated. In our quest for a booster antigen, we have studied Rv3097c of *M. tuberculosis* encoding a lipase (LipY) in a mouse model as a protective antigen to counter infection of *M. tuberculosis* (4, 5).

When the Rv3097c gene was overexpressed from a plasmid in BCG, the recombinant BCG lost immunogenicity. That is, mice immunized with recombinant BCG and challenged with *M. tuberculosis* were sensitive to killing just like naïve, unimmunized mice. We found over-expression of LipY caused suppression of the protective host immune response (Th1) and the rise of the immunosuppressive Th2 response (4). Mice died rapidly with reduced expression of cytokines and interleukins (4). Similarly, when Rv3097c gene was overexpressed in *M. tuberculosis*, recombinant *M. tuberculosis* was more virulent than wild-type *M. tuberculosis*. The mean survival time of infected mice was reduced, the bacillary load was higher, and lung pathology was severe. However, mice immunized with recombinant, purified LipY were protected against challenge with *M. tuberculosis*, and this correlated with an effective immune response (5). As the LipY lipase is a cell wall-associated protein that interacts with effector molecules of the immune system (6), immunization with subunit antigen LipY could generate an immune response against mycobacterial lipase. LipY and other similar lipolytic enzymes could therefore be explored as potential adjuncts to BCG or new therapeutic vaccine candidates against mycobacterial infections (Figure 1) (5).

**Figure 1:**
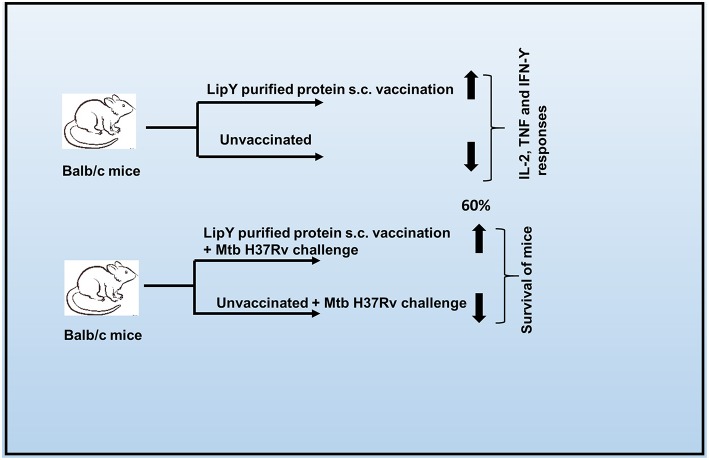
Potential role of LipY as a vaccine candidate for protection against tuberculosis.

Non-tubercular mycobacteria (NTM) have also been known to cause a spectrum of diseases in humans, although with less severe pathogenesis as compared to *M. tuberculosis*. *M. fortuitum*, which is an NTM, has been historically shown to cause opportunistic secondary infections in humans. Mice are susceptible to infection of *M. fortuitum*, and display disease symptoms, but infection only causes mortality in 25–30% of cases. Bacilli lodge in the kidney, where they multiply and cause symptoms (7). Bioinformatic analysis indicates that the *M. fortuitum* genome has no Rv3097c gene or its homolog as confirmed by sequence analysis and western blot (unpublished data). When Rv3097c gene was expressed in *M. fortuitum*, mortality of mice infected with recombinant *M. fortuitum* increased to 100% compared to 25–30% with wild-type *M. fortuitum* infected mice. Thus, LipY lipase can modulate the virulence of *M. tuberculosis* and NTM *M. fortuitum* by downregulating the host immune response.

The published literature and our analyses suggest that mycobacterial LipY lipase is a cell wall-associated enzyme, which also acts as a virulent factor in *M. tuberculosis*. LipY lipase has an important function in the biology of mycobacteria. It causes catabolism of stored triacylglycerol, thus releasing free fatty acids as a “lipid diet” for starving mycobacteria during latency (8, 9). LipY is up-regulated in various conditions and environments. We measured gene expression of LipY using real-time PCR in various *in vitro, ex vivo*, and *in vivo* conditions. LipY expression was enhanced in infected mouse macrophages (10) and in the lungs of infected mice (11). An increase in expression of LipY was observed in cells grown *in vitro* in oxygen and nutrient-depleted conditions that mimic dormancy (12). Therefore, environmental stress induces an increase in transcription of the LipY gene (Rv3097c). Interestingly, inhibitors of LipY were identified in our lab that inhibited the growth of the bacilli under hypoxic conditions, but not that of aerobically-grown cultures (13).

In conclusion, there has been much effort invested in augmenting the BCG vaccine, with various and often disappointing results. The LipY lipase is an important antigen of *M. tuberculosis* that could be explored to boost the BCG vaccine and improve protection against TB.

## Author Contributions

BS wrote the manuscript and VS edited the manuscript. VK, RS, AK, and CJ provided the suggestions and comments to improve the manuscript.

### Conflict of Interest Statement

The authors declare that the research was conducted in the absence of any commercial or financial relationships that could be construed as a potential conflict of interest.
